# Predictive factors for prognosis after gastrostomy placement in routine non-invasive ventilation users ALS patients

**DOI:** 10.1038/s41598-020-70422-2

**Published:** 2020-09-15

**Authors:** Adèle Hesters, Maria del Mar Amador, Rabab Debs, Nadine Le Forestier, Timothée Lenglet, Pierre-François Pradat, François Salachas, Morgane Faure, Maria-Alejandra Galarza Jimenez, Jesus Gonzalez-Bermejo, Capucine Morelot, Gaëlle Bruneteau

**Affiliations:** 1 Département de Neurologie, Centre de Référence SLA, Centre de Recherche en Myologie, UMRS974, APHP, Hôpital Pitié-Salpêtrière, 47-83 Bd de l’Hôpital, 75013 Paris, France; 2 Département de Recherche en Ethique, Université Paris Sud/Paris Saclay EA 1610 : Etude des sciences et techniques, Pr Emmanuel Hirsch, Paris, France; 3 Laboratoire D’Imagerie Biomédicale, CNRS, INSERM, Sorbonne Université, Paris, France; 4 Northern Ireland Centre for Stratified Medicine, Biomedical Sciences Research Institute Ulster University, C-TRIC, Altnagelvin Hospital, Londonderry, UK; 5 Service de Pneumologie, Médecine Intensive Et Réanimation, APHP, Hôpital Pitié-Salpêtrière, Paris, France; 6 Unité Fonctionnelle SSR Respiratoire, APHP, Hôpital Pitié-Salpêtrière, 75013 Paris, France; 7 UMRS 1158, Neurophysiologie Respiratoire Expérimentale Et Clinique, Sorbonne Universités - Inserm, Paris, France; 8 Institut National de La Santé Et de La Recherche Médicale, Association Institut de Myologie, Centre de Recherche en Myologie, UMRS974, Sorbonne Université, Paris, France

**Keywords:** Amyotrophic lateral sclerosis, Respiratory signs and symptoms

## Abstract

Due to the expanding use of non-invasive ventilation (NIV) in amyotrophic lateral sclerosis (ALS), the question of enteral nutrition is increasingly raised in NIV users ALS patients. Here, we aimed to determine the prognostic factors for survival after gastrostomy placement in routine NIV users, taking into consideration ventilator dependence. Ninety-two routine NIV users ALS patients, who underwent gastrostomy insertion for severe dysphagia and/or weight loss, were included. We used a Cox proportional hazards model to identify factors affecting survival and compared time from gastrostomy to death and 30-day mortality rate between dependent (daily use ≥ 16 h) and non-dependent NIV users. The hazard of death after gastrostomy was significantly affected by 3 factors: age at onset (HR 1.047, *p* = 0.006), body mass index < 20 kg/m^2^ at the time of gastrostomy placement (HR 2.012, *p* = 0.016) and recurrent accumulation of airway secretions (HR 2.614, *p* = 0.001). Mean time from gastrostomy to death was significantly shorter in the dependent than in the non-dependent NIV users group (133 vs. 250 days, *p* = 0.04). The 30-day mortality rate was significantly higher in dependent NIV users (21.4% vs. 2.8%, *p* = 0.03). Pre-operative ventilator dependence and airway secretion accumulation are associated with worse prognosis and should be key decision-making criteria when considering gastrostomy tube placement in NIV users ALS patients.

## Introduction

Malnutrition and weight loss are independent negative prognostic factors for survival in Amyotrophic Lateral Sclerosis (ALS)^1^. In case of severe dysphagia and/or malnutrition, gastrostomy placement (GP) is frequently offered to maintain adequate nutritional intake in these patients. Optimal timing for GP remains ill-defined and current guidelines^1,2^ recommend to take into account the severity of dysphagia, malnutrition, respiratory function (vital capacity, VC > 50%) and the patient’s general condition. Early insertion of a feeding tube is recommended^2^ but GP is at times delayed because of patient’s reservations about enteral nutrition. A recent large prospective study^3^ identified two main prognostic factors after GP: age at onset of ALS and nutritional status (evaluated by the percentage of weight loss from diagnosis to gastrostomy). In this study, 25% of the patients were routine users of non-invasive ventilation (NIV) but specific prognostic factors in this subpopulation were not studied. However, due to the increasing use of NIV in the ALS population, the question of GP in routine NIV users with ventilator dependence often arises in clinical practice. Here, we aimed to determine the prognostic factors after GP in routine NIV users, taking into account NIV use daily duration and the amount of airway secretions evaluated by the number of clearance treatment sessions required per day.

## Patients and methods

### Patients and data collection

**Figure 1:**
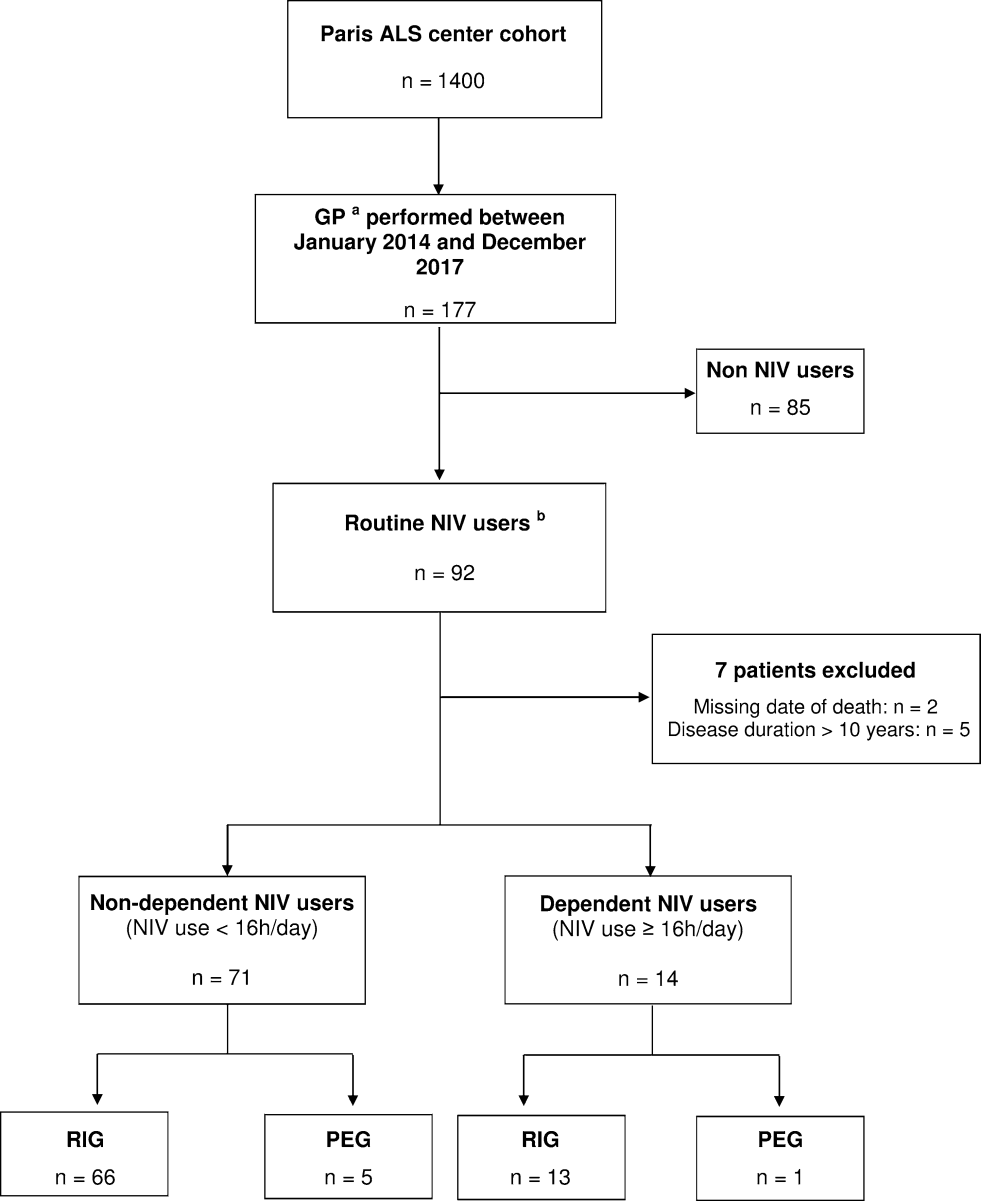
Flowchart of patients included in the study. ^a^The indications for GP were a reduction of 5% or more of pre-onset weight, and/or severe dysphagia. The method of insertion was decided by the referent neurologist, RIG procedure being preferred for patients with a more compromised respiratory function. ^b^The NIV settings were systematically optimized if necessary within 48 h before GP. In patients with regular NIV use ≥ 16 h/day, GP was performed under NIV. For patients with nocturnal ventilation or up to 16 h per day, NIV was used if respiratory discomfort and/or oxygen desaturation occurred during the procedure. ALS: amyotrophic lateral sclerosis, GP: Gastrostomy placement, NIV: non-invasive ventilation, RIG: radiologically-inserted gastrostomy, PEG: percutaneous endoscopic gastrostomy.

Between January 2014 and December 2017, 92 patients with a diagnosis of definite, probable, laboratory supported or possible ALS (according to the revised El Escorial criteria^4^) and who were routine NIV users, underwent GP at the Paris ALS center, France (Fig. 1). The indications for GP were a reduction of 5% or more of pre-onset weight, and/or severe dysphagia. The method of insertion—percutaneous endoscopic gastrostomy (PEG) or radiologically-inserted gastrostomy (RIG)—was decided by the referent neurologist^5^. RIG procedure was preferred for patients with a more compromised respiratory function whereas PEG procedure was usually chosen for patients at a less advanced stage of the disease, who were predicted to be able to tolerate endoscopy (able to flat, receive sedation)^6^. Whichever method of gastrostomy insertion was chosen, the NIV settings were systematically checked and optimized if necessary within 48 h before GP. The effectiveness of NIV was assessed with arterial blood gases, analysis of ventilator-recorded tracings and nocturnal oximetry. In patients with regular NIV use ≥ 16 h/day, GP was performed under NIV. For patients with nocturnal ventilation or up to 16 h per day, NIV was used if respiratory discomfort and/or oxygen desaturation occurred during the procedure. Oxygen supplementation into the ventilator was not routinely necessary. For RIG, premedication (with sedatives or narcotics) was not required but local anesthesia (lidocaine) was performed to prevent pain. Emergency airway equipment and an anesthesiologist were immediately available if needed. Patients were followed up every three months after GP. The following data were retrospectively collected from patients’ medical records: gender, age at onset, site of onset, method of GP, revised ALS Functional Rating Scale (ALSFRS-R) score, date of death or start of invasive mechanical ventilation (IMV), body mass index (BMI) at the time of GP, daily duration of NIV (< or ≥ 16 h/day), and amount of airway secretions. We estimated the amount of airway secretions by the number of airway clearance sessions (with mechanical insufflator-exsufflator) required per day during the hospitalization for the GP (“absent” or “occasional” when less than 1 session per day was needed, “recurrent accumulation” when at least 1 session per day was needed). Patients were admitted to the hospital a few days before gastrostomy placement, allowing quantification of airway secretions before gastrostomy insertion. Cough efficacy was systematically evaluated on hospital admission, and a cough assist device was available for all patients. Audible airway sounds with breathing (generated by secretions), with or without decreased oxygen saturation level and/or dyspnea, were the main criteria used to indicate the need for an airway clearance session.

The cutoff time for follow-up was Oct 31, 2018. Five patients with disease duration > 10 years and two dead patients in whom the exact date of death was unknown were excluded from analysis. This retrospective study was performed in agreement with local ethical regulations and approved by the French National Data Protection Authority (registration number 2201855). It was performed in accordance with relevant guidelines and regulations and informed consent was obtained for GP in all patients.

### Statistical analysis

Statistical analyses were conducted using GraphPad Prism 6 and Microsoft®Excel XLSTAT 2018.5. Mann–Whitney test was used for quantitative variables and Fischer’s test for qualitative variables. Survival was assessed using Kaplan–Meier curve. Cox proportional hazards regression was used to examine factors affecting survival after GP. As covariates, we included previously reported factors (age at disease onset^3,7,8^ and site of onset^9^) and BMI at the time of GP (< or ≥ 20 kg/m^2^) as a marker of nutritional status. Based on clinical judgment, we also included daily duration of NIV and amount of airway secretions. The level of significance was set at 0.05.

## Results

Baseline demographic and clinical characteristics of the patients are shown in Table 1. There were no differences between dependent NIV users (≥ 16 h/day) and non-dependent patients (nocturnal ventilation or less than 16 h/day) in terms of age and site of disease onset, disease duration at the time of the procedure, method of GP, BMI and amount of airway secretions (Table 1). Overall median survival was 231 days. At the end of follow-up, 73 patients (87%) had died and one patient (in the non-dependent NIV user group) had started IMV 81 days after GP. The overall 30-day mortality rate was 5.9% (5 patients). Cox regression analysis showed that the hazard of death after gastrostomy in routine NIV users was significantly affected by 3 factors (Table 2): age at onset (HR 1.047 [95%CI 1.013–1.083]; *p* = 0.006), BMI measured at the time of GP (lower *vs* higher than 20 kg/m^2^, HR 2.012 [95%CI 1.141–3.545]; *p* = 0.016) and recurrent accumulation of airway secretions (HR 2.614 [95%CI 1.481–4.613]; *p* = 0.001). There was a trend towards a 1.8 increased risk of death in dependent NIV user patients, which did not reach statistical significance. To further investigate this aspect, we analyzed the time from gastrostomy to death or IMV (TTD). The mean TTD after gastrostomy was significantly shorter in the dependent than in the non-dependent NIV users group (133 days [range 2–348] vs. 250 days [range 18–1024], *p* = 0.04). Finally, the 30-day mortality rate was significantly higher in dependent NIV users than in non-dependent patients (respectively 3/14 [21.4%] and 2/71 [2.8%] patients, *p* = 0.03).

**Table 1:** Baseline demographic and clinical characteristics of the patients.

Parameter	All patients, n = 85	Non-dependent NIV users, n = 71	Dependent NIV users, n = 14	*p*
Gender: Male (n, %)	42 (49%)	34 (48%)	8 (57%)	0.46
Age at disease onset (years)	60.3 [17–88]	60.3 [17–80]	59.8 [39–88]	0.47
Site of disease onset: Bulbar (n, %)	44 (52%)	39 (55%)	5 (36%)	0.19
Disease duration from onset to gastrostomy insertion (months)	32.4 [7–87]	33.1 [7–87]	29 [17–46]	0.61
Type of gastrostomy insertion (n, %)				
PEG	6 (7%)	5 (7%)	1 (7%)	> 0.99
RIG	79 (93%)	66 (93%)	13 (93%)	
ALSFRS-R score	21.6 [10–45]	22.4 [10–45]	16.8 [14–29]	0.08
BMI (kg/m^2^)	19.1 [13.6–26.5]	19.2 [13.6–26.5]	18.7 [15–23.4]	0.63
Amount of airway secretions (n, %)^a^				
Absent or occasional	48 (63%)	42 (67%)	6 (46%)	0.21
Recurrent accumulation	28 (37%)	21 (33%)	7 (54%)	
FVC (% predicted value)	38 [14–68]	38.8 [14–68]	34.5 [23–47]	0.80
PaCO_2_ (mmHg)^b^	42.3 [27.1–51.6]	42.8 [27.1–51.6]	39.8 [31.5–50.4]	0.06
Dead at the end of follow-up (n, %)^c^	73 (86%)	61 (86%)	12 (86%)	> 0.99
Dead within 30 days after gastrostomy^d^	5%	2 (2.8%)	3 (21.4%)	0.03
Time from gastrostomy to death or IMV (days)^e^	235 [2–1024]	250 [18–1024]	133 [2–348]	0.04
Total length of hospital stay (days)	16.5 [5–60]	15.8 [5–60]	20.1 [8–43]	0.03

Data are expressed in n (%) or mean [range].

NIV: non-invasive ventilation, PEG: percutaneous endoscopic gastrostomy, RIG: radiologically-inserted gastrostomy, ALSFRS-R: revised ALS Functional Rating Scale, collected at the time of GP or up to the previous 3 months (n = 29, including 13 patients in the non-dependent NIV group), BMI: body mass index (n = 83, including 71 patients in the non-dependent NIV group), FVC: forced vital capacity collected up to the previous 6 months before GP (n = 23, including 19 patients in the non-dependent NIV group), with mean time from last available FVC to gastrostomy = 96 days in all patients, 98 days in the non-dependent NIV group and 85 days in the dependent NIV group, PaCO2: arterial carbon dioxide pressure (n = 85), IMV: Invasive Mechanical Ventilation.

^a^n = 76, including 63 patients in the non-dependent NIV group. The amount of airway secretions was estimated by the number of airway clearance sessions (with mechanical insufflator-exsufflator) required per day during the hospitalization for the GP: “absent” or “occasional” when less than 1 session per day was needed, “recurrent accumulation” when at least 1 session per day was needed.

^b^Arterial blood gases were collected early in the morning while on NIV.

^c^The most common cause of death was respiratory failure in 45 (61.6%) patients including 6 patients (8.2%) with pulmonary infection. Sudden cardiac death was reported in 8 patients (11%). The cause of death was unknown in 20 patients (27.4%).

^d^In the NIV dependent group, the causes of early death (within 30 days) were pneumonia (n = 1), respiratory insufficiency (n = 1) and unknown for one patient. In the non-dependent NIV patients, the causes of early death were respiratory insufficiency for one patient and sudden death for one patient.

^e^n = 74, 62 patients in the non-dependent NIV group including one patient with IMV.

**Table 2:** Multivariate analysis using Cox proportional hazard regression model.

Variable		Hazard ratio	p value	95% CI
Age at onset	Older versus younger	1.047	0.006	1.013–1.083
Disease-onset region	Spinal versus bulbar onset	1.122	0.695	0.632–1.989
BMI	Lower versus higher than 20 kg/m²	2.012	0.016	1.141–3.545
Daily duration of NIV	More versus less than 16 h/day	1.824	0.132	0.835–3.985
Amount of airway secretions	“Recurrent accumulation” versus “absent or occasional”	2.614	0.001	1.481–4.613

BMI, body mass index, NIV, non-invasive ventilation.

## Discussion

In accordance with previous studies, we found that the hazard of death after GP in routine NIV users ALS patients was significantly affected by the age at onset and nutritional status^3,8^. In addition, we showed that, in this particular ALS patient population, preoperative respiratory status strongly influences prognosis. Indeed, recurrent accumulation of airway secretions (requiring at least one airway clearance session per day) was associated with a 2.6 increased risk of death after GP. Airway secretion accumulation is known to be associated with poor tolerance of NIV in ALS patients^10^. Our study shows that recurrent accumulation of airway secretions significantly worsens prognosis after GP in routine NIV users ALS patients, highlighting that adequate peri-operative management of airway secretions is crucial in this context.

Pre-operative NIV dependence (defined by NIV daily use ≥ 16 h/day) was also associated with a worst prognosis after GP, with a significantly higher 30-day mortality rate and lower TTD. Although the hazard of death tended to increase with NIV dependence, it did not reach statistical significance in the Cox regression analysis. This may be due to the high cut-off value for NIV use duration that we used to define “ventilation dependence”. There is no clear definition of a ventilator-dependent patient^11^. The cut-off of 16 h/day corresponds to the “life support ventilation” category defined by the French National Authority for Health, in which an external battery pack and a back-up ventilator must be provided to the patient^12^. However, patients unable to remain 12 h/day in spontaneous ventilation may already be considered as ventilator-dependent^13^. The threshold above which NIV dependence is associated with an increased risk of death after GP may thus be lower than 16 h/day. If NIV use per se does not seem to have an effect on survival after GP^7,8,14^, there are almost no data about the impact of daily NIV use duration on prognosis. One study found no significant difference in survival between patients under “continuous” NIV (i.e. who could not be weaned from ventilator) when compared to patients using it intermittently^15^. However, NIV use daily duration in the non-permanent users was not specified even though “intermittent” use encompasses very different clinical situations. Overall, our results highlight that GP should be discussed early enough in NIV users ALS patients, when ventilator daily use duration increases with a need for significant diurnal ventilation.

Our study has limitations. First, data were collected retrospectively and in a single center. In the context of impaired respiratory function, RIG insertion was chosen for most (93%) of the patients, which did not allow us to compare the effect on survival of the different insertion methods. The retrospective nature of the study also did not allow us to collect more precisely the NIV daily use duration for each patient, but only to classify patients as non ventilator-dependent or ventilator-dependent (i.e. daily use ≥ 16 h). We evaluated the amount of airway secretions by the number of clearance treatment sessions required per day—a parameter likely to be available in routine practice for most patients—but the exact volume of mucus expelled was not precisely quantified. Finally, our study did not evaluate the effect of gastrostomy on quality of life. However, the potential impact of GP on quality of life is an important parameter to consider, in the context of increasing ventilation dependence and motor disability.

Keeping those limitations in mind, the results of our study point out that pre-operative ventilator dependence and recurrent accumulation of airway secretions are key prognosis factors after GP in routine NIV user ALS patients. In addition to well-known prognosis factors (age and nutritional status), these two respiratory parameters should be evaluated when considering GP in NIV users ALS patients.

## Conclusion

Pre-operative ventilator dependence and airway secretion accumulation are associated with worse outcome after gastrostomy insertion in NIV users ALS patients and should be taken into consideration in the decision-making process.

## Data Availability

The data supporting this study will be made available upon reasonable request.
